# Bedside Assessment of Tissue Oxygen Saturation Monitoring in Critically Ill Adults: An Integrative Review of the Literature

**DOI:** 10.1155/2014/709683

**Published:** 2014-05-08

**Authors:** Carol Diane Epstein, Karen Toby Haghenbeck

**Affiliations:** ^1^College of Health Professions, Lienhard School of Nursing, Pace University, Office 319, 861 Bedford Road, Pleasantville, NY 10570, USA; ^2^College of Health Professions, Lienhard School of Nursing, Pace University, Office L308, 861 Bedford Road, Pleasantville, NY 10570, USA

## Abstract

*Objective*. Tissue oxygen saturation (StO_2_) monitoring is a noninvasive technology with the purpose of alerting the clinician of peripheral hypoperfusion and the onset of tissue hypoxia. This integrative review examines the rigor and quality of studies focusing on StO_2_ monitoring in adult critically ill patients. * Background*. Clinicians must rapidly assess adverse changes in tissue perfusion while minimizing potential complications associated with invasive monitoring. The noninvasive measurement of tissue oxygen saturation is based on near-infrared spectroscopy (NIRS), an optical method of illuminating chemical compounds which absorb, reflect, and scatter light directed at that compound. * Methods*. An integrative review was conducted to develop a context of greater understanding about complex topics. An Integrative review draws on multiple experimental and nonexperimental research methodologies. * Results*. Fourteen studies were graded at the C category. None reported the use of probability sampling or demonstrated a cause-and-effect relationship between StO_2_ values and patient outcomes. * Conclusions*. Future research should be based on rigorous methods of sampling and design in order to enhance the internal and external validity of the findings.

## 1. Introduction

Tissue oxygen saturation (StO_2_) monitoring is a relatively new technology that has been reported to function as an early warning sign of peripheral hypoperfusion and the onset of tissue hypoxia [1–4]. Also called a tissue spectrometer, StO_2_ technology is based on near-infrared spectroscopy (NIRS), an optical method of illuminating chemical compounds which absorb, reflect, and scatter light directed at that compound. NIRS technology has its origins a century ago, and its application to the human body was developed in the 1970s by Jobsis for the invasive monitoring of cerebral and cardiac oxygenation [5]. In the case of StO_2_ monitoring, the measured chromophore compounds include oxyhemoglobin, deoxyhemoglobin, and total hemoglobin concentrations in the muscle tissue bed. The underlying rationale for bedside StO_2_ monitoring is its potential capacity to alert the clinician that peripheral blood flow is being redistributed to vital organs as the normal balance between the proportions of oxyhemoglobin and deoxyhemoglobin in the peripheral tissues undergoes adverse changes. Initially used as a marker of the adequacy of resuscitation in patients with hemorrhagic shock, StO_2_ monitoring has attracted interest because of its noninvasive and real time nature, as well as its potential utility in research efforts designed to reduce morbidity and mortality [6, 7]. The purpose of this integrative review is to review the quality and rigor of studies employing StO_2_ monitoring at the bedside in the intensive care unit (ICU).

## 2. Methods

The methodology for this integrative review is based on the model recommended by Whittemore and Knafl [8]. This approach draws on both experimental and nonexperimental research as well as theoretical, historical, qualitative, methodological, and expert empirical perspectives from the scientific literature in order to develop a context of greater understanding about a complex topic of concern. The construct of tissue hypoxia seems well suited to this type of review. After more than a century of research in multiple disciplines, definition and validation of the phenomenon remain elusive, complex, and difficult to operationalize in research studies. For example, in a study of peripheral vasoconstriction on StO_2_ values in human volunteers, Lima and colleagues recently cautioned that the effect of thenar skin circulation on StO_2_ signals may mislead the clinician into concluding that tissue hemoglobin is undergoing oxygen desaturation when it is not [9].

Whittemore and Knafl proposed several strategies to enhance rigor and avoid subjective bias in the development of an integrative review. During the* problem identification stage*, the variables of interest should be specified, for example, concepts, target population, and health care problem. The scientific literature related to the broad topic of NIRS consists of at least 60,000 studies and reviews. For the purpose of this review, the concepts of interest are focused on the following problem statement: what is the quality of the scientific evidence on StO_2_ data in critically ill adults, generated by the technology of near-infrared spectroscopy (NIRS)?

The next step of an integrative review is to select an appropriate sampling frame, for example, empirical studies, inclusion of theoretical literature, historical perspectives, terms used in the literature search, the databases used, additional search strategies, identification of articles in reference lists, and the inclusion and exclusion criteria for determining relevant primary sources. A search for articles published in English from 1996 to 2013 and indexed in CINAHL, MEDLINE, Joanna Briggs Institute, and Google Scholar databases was performed using the following key terms:* tissue hypoxia, tissue oxygenation saturation (StO*_*2*_ *), critically ill adults in the intensive care unit (ICU)*,* serum lactate, mortality, *and* multiple organ dysfunction syndrome (MODS)*. Studies which met at least four of these criteria were included for review. The literature search focused specifically on studies consisting of sample populations of critically ill adult patients. As recommended by Whittemore and Knafl, citations in retrieved journal articles were examined for possible omission of relevant articles. Research abstracts were not considered for review. Exclusion criteria consisted of studies using NIRS monitoring of pediatric patients and patients receiving cerebral oxygenation monitoring, as these sampling frames were not consistent with our specific topic of interest. The authors independently reviewed and graded each study, using the American Association of Critical-Care Nurses' (AACN) Evidence-Leveling Hierarchy (Table 1) [10]. When the authors shared their ratings, they continued discussion until consensus was reached.

## 3. Background

### 3.1. Near-Infrared Spectroscopy: A Method for Monitoring Tissue Oxygen Saturation

Near-infrared spectroscopy (NIRS) is an optical technology which has been exploited for monitoring tissue oxygen saturation at the bedside [11]. In his comprehensive review, Chance dates the origins of spectroscopy, which he called the “optical method,” to Otto Warburg in the 1930s [12]. Spectroscopy was used to study alterations in the light-absorbing and light-reflecting characteristics of substrates as they undergo chemical changes. As light is directed at an organic compound in the body, such as adenosine triphosphate (ATP), the compound absorbs that light and loses its intensity at a known rate, known as attenuation. Therefore, the identity and the concentration of an unknown compound can also be quantified based on known characteristics. Pioneering work in NIRS spectroscopy by Chance and Williams using reflectance probes, then called* reflectance spectrophotometry*, produced descriptions of differential optical light-absorbing characteristics of cytochromes in the respiratory chain during normal oxidative phosphorylation as well as during conditions of hypoxia and anoxia on isolated mitochondria [13]. At this point in history, scientists had devised dual wavelength monitoring to monitor oxygen uptake when cytochromes were exposed to variations in oxygen availability and adenosine diphosphate (ADP) concentration. The next large step was to utilize NIRS to study cytochrome function in intact brains of awake, whole animals and to probe whether there was a critical PO_2_ at which point ATP synthesis declines irreversibly [14]. The original application of NIRS to the human body was developed by Jobsis in the 1970s, specifically in the monitoring of cerebral and cardiac oxygenation [15]. When NIRS is used to study biological tissues, such as the chromophore hemoglobin, data generated by this technology is calculated by the differences in the light-absorbing and light-reflecting characteristics of oxyhemoglobin and deoxyhemoglobin, which in turn provide information about changes in hemoglobin concentration [5]. Chance referred to spectroscopic analysis of changes in hemoglobin concentration as “tissue hemoglobinometry” [12].

Across the full electromagnetic spectrum (EMS) of light, visible light and our ability to see color lie in the mid-spectrum range of 400 to 700 nanometers (nm) [16]. The near-infrared light spectrum ranges from 700 nm to 1000 nm, above (or visually speaking, to the right of) visible light on the EMS spectrum, and the wavelengths approximately 650 to 900 nm are used in the clinical applications of NIRS technology. The NIRS technology is primarily used to measure regional cerebral oxygen saturation (rSO_2_). Near-infrared light is able to access up to a tissue depth of 8 centimeters (cm), whereas visible light can penetrate no deeper than one cm [5]. The NIRS instrumentation light source is a laser diode. There are currently 3 types of spectrometers: continuous wave, time-of-flight, and frequency domain. Continuous wave NIRS is the most commonly used noninvasive spectrometer in research and clinical settings. The instrumentation consists of at least 3 wavelengths, but most devices have 4 in order to differentiate more accurately among the different types of hemoglobin. This type of NIRS is used in measurement of neonatal cerebral blood flow and volume with an oxygen tracer. Cerebral venous oxygenation can also be measured with NIRS by several maneuvers, for example, gentle occlusion of the jugular vein, manipulation of cerebral blood volume by altering ventilation, or by gravity, as the infant's body is shifted to increase venous blood volume. Beyond the neonatal ICU, NIRS has been used to measure regional tissue oxygen saturation in many types of conditions, including adult non-ICU patients who were (1) undergoing esophageal and colorectal cancer surgery [17, 18]; (2) at risk for postoperative abdominal surgical infection [19]; and (3) experiencing intermittent claudication [20].

Noninvasive measurement of skeletal muscle tissue oxygen saturation of hemoglobin (StO_2_) by NIRS is based on wavelength analysis of 3 differential forms of hemoglobin, including oxyhemoglobin, deoxyhemoglobin, and total hemoglobin [21]. The spectrometer is programmed with an algorithm derived from 4 wavelengths of light at 680, 720, 760, and 800 nanometers (nm), respectively, reflecting the interaction of optical light paths absorbed by chromophores, including the following: (1) oxyhemoglobin concentration [HbO_2_]; (2) deoxyhemoglobin concentration [HHb]; (3) total hemoglobin concentration [HbO_2_] + [HHb]; and (4) StO_2_, tissue hemoglobin oxygen saturation [HbO_2_]/([HbO_2_]+ [HHb]). The final StO_2_ percentage value is displayed on a monitor.

The StO_2_ light probe is most often placed adhesively on the thenar eminence of the hand, that is, the thumb muscle. Normal StO_2_ values in human volunteers have been reported to average 87 ± 6% in two separate studies [22, 23]. Under conditions of intact autoregulation, the peripheral microcirculation in the muscle should vasoconstrict during the onset of shock in order to shunt blood flow to critical organs; theoretically, a decrease in StO_2_ should be displayed on the monitor immediately [24–26]. Clinicians who work in intensive care, the emergency department, operating suites, and postanesthesia care would have bedside access to this information while invasive devices are placed. In order to evaluate the scientific literature on the clinical utility of StO_2_ monitoring, we conducted a review of the literature focusing on the use of the StO_2_ monitoring in studies of high acuity and critically ill patients.

### 3.2. Review of the Literature: Results

There are 3 types of spectrometers cited in the literature search: (1) the EQUANOX Model 7600 Regional Oximetry System (Nonin Medical Inc., Plymouth, MN, USA), (2) the* InSpectra* tissue spectrometer (Hutchinson Technology Inc., BioMeasurement Division, Hutchinson, MN, USA), and (3) the INVOS 5100C Cerebral/Somatic Oximeter (Covidien, Dublin, Ireland). The EQUANOX Model 7600 Regional Oximetry System has been used in studies of outpatient problems such as sleep apnea and among community patients subjected to high altitude conditions. The INVOS 5100C Cerebral/Somatic Oximeter was specifically applied to neonatal patients and head-injured patients receiving NIRS monitoring of cerebral oxygenation. The* InSpectra* tissue spectrometer was the only device used for ICU adult patients at risk for tissue hypoxia.

The American Association of Critical-Care Nurses' (AACN) Evidence-Leveling Hierarchy (Table 1) [10] was used to evaluate the quality and rigor of the study designs. A literature search identified 14 studies for final analysis (Table 2). Review of each study was carried out independently by the authors (Carol Diane Epstein and Karen Toby Haghenbeck) and then mutually by an iterative process. All studies were leveled at the C category and described by the AACN's Evidence-Based Practice Work Group as “qualitative studies, descriptive or correlational studies, integrative reviews, systematic reviews, or randomized controlled trials with inconsistent results” [10]. All study designs were characterized as observational, retrospective, correlational, or prospective in nature. None reported the use of probability sampling, random sampling of subjects, or random assignment of subjects. The study strengths and limitations were identified. Special attention was given to the degree of manufacturer support for the implementation of the research and whether mention was made of the interpretation of data by manufacturer representatives prior to publication.

### 3.3. Studies of Trauma Patients

Five studies of injured patients utilized prospective, retrospective, and observational designs in which a predetermined StO_2_ cutoff value functioned as the intervening variable and patient outcomes such as multiple organ dysfunction syndrome (MODS) and mortality served as dependent variables (Table 2). Beilman and colleagues [27] conducted a multicenter, retrospective, post hoc analysis of 356 patients (Table 2, Study 1) enrolled in a larger study (Table 2, Study 3) [7]. In the post hoc analysis, the effect of hypothermia on patient outcome was examined, based on a StO_2_ cutoff value of 75%. Multivariate logistic regression analysis determined that the minimum StO_2_ (<75%) within the first hour of admission was one of several significant predictors of MODS (*P* = 0.0014) and mortality (*P* = 0.0021) in both normothermic and hypothermic patients. The minimum StO_2_ values are not reported, with the exception of confidence intervals of 74% (62% to 89%). Elevated base deficit was able to predict mortality (*P* = 0.0454), but not MODS, among both hypothermic and normothermic patients.

In the prospective study of the same patients (Table 2, Study 3), Cohn and coworkers sought to identify the threshold StO_2_ value for predicting MODS and mortality as well as the strength of its ability to predict these outcomes in 381 trauma patients [7]. A StO_2_ value of 75% was determined empirically from the data analysis. The positive predictive values for MODS and mortality were 18% and 20%, respectively. That is, StO_2_ values less than 75% accurately predicted MODS and patient death only 20% of the time. The negative predictive value is the probability that subjects who did not develop MODS or die truly did not; in this case, StO_2_ values performed well at 91% and 95%, respectively. The clinical implications from the findings of this negative predictive value suggest that StO_2_ values greater than 75% indicate the absence of tissue hypoperfusion and, possibly, tissue hypoxia. Yet the positive predictive value of only 20%, when StO_2_ values fall below 75%, suggests that the possible causes and interpretation of data are either inaccurate, unclear, or subject to theoretical or technological error.

Cairns and coworkers [4] (Table 2, Study 2) were the first to investigate the use of NIRS in severely traumatized patients following early goal directed therapy based on supranormal oxygen transport goals within the first 12 hours of admission. They proposed a novel hypothesis of the relationship between early* oxygen supply-independent mitochondrial dysfunction* and MODS in trauma patients. In other words, these investigators hypothesize that the construct of oxygen supply-dependent oxygen consumption can be operationalized and identified at the level of the mitochondria during the onset of tissue hypoxia. In order to measure the “decoupling” oxidative changes in cytochrome a, a3, an additional fiber-optic probe was used to supply infrared light for the cytochrome's light absorption spectrum characteristics. However, the authors state that the underlying empirical support for the definition of “decoupling” was derived from unpublished data. The authors defined this event as occurring when the relative rates of change in absorption of oxyhemoglobin (HbO_2_) and cytochrome a, a3 no longer rise and fall in concert. Evidence of decoupling was found to be statistically significant in 8 (89%) of the patients who developed MODS, compared to 13% of patients who did not. Lactate levels were significantly higher in 9 (38%) who developed MODS (3.6 ± 0.3 versus 1.7 ± 0.2).

Ikossi and colleagues studied StO_2_ values in 28 trauma patients who were assessed to be adequately resuscitated by objective criteria following ICU admission (Table 2, Study 5) [28]. They also compared correlations between measurements of StO_2_ and the partial tissue pressure of oxygen in (PmO_2_), an invasive probe-equipped device inserted into the deltoid muscle. The authors propose that their findings established “normal” values of the mean StO_2_ values in fully resuscitated patients; however, the mean StO_2_ of 63% ± 27 is relatively low compared to findings subsequently reported in normal subjects [22, 23], while the standard deviation (SD) is large. Low StO_2_ values (<35%) persisting for longer than 2 hours on the first day of admission were able to predict MODS (odds ratio 12.8 [CI 1.3–131], *P* = 0.03). In spite of these significant results, the authors do not adequately address possible reasons why fully resuscitated patients should have such low StO_2_ values.

Smith and coworkers studied the utility of StO_2_ as a trigger for blood transfusion in 22 trauma patients using a cutoff StO_2_ value of 70% (Table 2, Study 14) [29]. The cutoff value was determined empirically by logistic regression following data collection. In this study, the StO_2_ value was operationalized as the independent variable, and blood transfusion was the dependent variable. StO_2_ measurements were made within one hour of admission; patients with low StO_2_ values were 9 times as likely to receive transfusion (*P* = 0.018). A strength of this study included blinding caregivers to the StO_2_ values, while the investigators acknowledge that physician bias for ordering blood transfusions may have occurred. Based on three deaths, findings from this study for the use of StO_2_ monitoring yielded a weak positive predictive value of 27% and a strong negative predictive value of 93%.

### 3.4. Studies of General Critically Ill Patients

Studies based on heterogeneous samples of ICU patients pose special challenges in interpretation of findings. In spite of this limitation, Lima et al. conducted a well-designed study by performing StO_2_ measurements in 22 critically ill patients with elevated serum lactate levels during the first 8 hours of ICU admission (Table 2, Study 8) [6]. All but three patients were diagnosed with varied shock states, including septic, cardiogenic, hypovolemic, hemorrhagic, and traumatic shock. A cutoff StO_2_ value of 70% was used to determine whether StO_2_ monitoring would demonstrate a relationship with the incidence of MODS and mortality. A major strength of this study was the blinding of the data collector to patient information and treatment. Among the 12 patients who at baseline had low StO_2_ values, ten patients were unable to normalize low StO_2_ values and were more likely to develop MODS, have an increased mortality, and have persistence of elevated serum lactate levels (*P* < 0.05).

### 3.5. Studies of Septic Patients

Studies of septic patients are inherently difficult to design and implement because the sampling start time frame may be difficult to identify [30]. The diagnostic criteria for the Surviving Sepsis Campaign, for example, are applied to the conditions of sepsis, severe sepsis, sepsis-induced hypotension, septic shock, and sepsis-induced hypoperfusion. Researchers must address the need to define* a priori* the inclusion/exclusion criteria as well as the time frames for study entry. For example, should one initiate the study early in the septic process or delay until resuscitation is thought to be complete? Three studies of septic patients were based on StO_2_ measurements (Table 2, Studies 7, 9, and 10). Leone and colleagues conducted a retrospective, observational study of 42 consecutive patients diagnosed with septic shock (Study 7) [31]. StO_2_ measurements were initiated when augmentation of global hemodynamic variables “seemed optimal, according to the decision of the attending physician (page 367).” Thus, the start time frame is unclear, and the duration of monitoring also remains uncertain. Overall, StO_2_ values were significantly lower in nonsurvivors than in survivors (73% [68–82%] versus 84% [81–90%], *P* = 0.02). A receiver operating curve analysis confirmed that StO_2_ was associated with mortality with an area under the curve at 71% (52–91%, *P* = 0.03), whereas elevated lactate levels were not found to be significantly related to patient death.

Mesquida and coinvestigators tested the potential relationship between oxygen delivery (DO_2_) and StO_2_ values in 15 septic patients who were enrolled in the study within 24 hours of demonstrating early signs of sepsis and following a decision to place a pulmonary artery catheter (PAC) (Study 9) [32]. The timing of measurements was not specified. While the correlation between DO_2_ and StO_2_ was fairly strong at 0.78 (*P* = 0.001), there was no significance detected for either value with the incidence of mortality. An interesting finding was the magnitude of the relationship between StO_2_ and invasive measurements of SvO_2_ (0.77, *P* = 0.001) and CvO_2_ (0.8, *P* = 0.001), thereby suggesting that StO_2_ could serve as a proxy for estimating mixed venous oxygen saturation of hemoglobin.

Mulier and colleagues also investigated the degree to which global invasive oxygen transport measurements and severity of sepsis correlated with StO_2_ values (Study 10) [33]. Inclusion criteria were strictly defined, as was timing of measurements. Caregivers were blinded to the StO_2_ data. The correlations between NIRS-related data and SvO_2_ were found to be statistically significant, but weak (*P* = 0.267). The relationship to patient outcome was not analyzed. Measurements of StO_2_, serum lactate, hemoglobin, and oxygen consumption (VO_2_) were also carried out in 9 healthy volunteers; however, the StO_2_ values were reported in general terms in which they were “higher” than in septic patients (page 517).

### 3.6. Methodological Studies

Emergence of new technologies commonly spawns innovative exploration of potential uses that were not initially associated with the device. In physiologic studies, the* validity* of measurements is commonly referred to as the accuracy of the phenomenon of interest, in this case, tissue hypoxia. The reliability of measurements, otherwise known as precision, quantifies whether the difference between two measurements separated by a time interval reflects a real change. Methodological studies often examine the accuracy and precision of measurements, as well as the degree to which intervening variables may interfere with them.

The development of third-space interstitial edema among septic patients as well as its influence on the variability of StO_2_ measurements was examined by Poeze (Table 2, Study 11) [34]. In order to explore which muscle sites are most accurate for measuring StO_2_,* InSpectra* sensors were adhesively placed in 8 septic patients at multiple muscle sites: bilateral deltoid muscles, bilateral brachial, the thenar eminence, and bilateral vastus and gastrocnemius muscles. Tissue thickness and the degree of edema were measured by echocardiography. The degrees of edema and skin thickness were found to be the lowest in the thenar eminence. StO_2_ values significantly correlated with degree of edema (*r* ^2^ = −0.44, *P* < 0.0001) and with total tissue thickness from skin to muscle (*r* ^2^ = −0.64, *P* = 0.0001).

Vascular occlusive testing (VOT) is used to help identify tissue hypoperfusion and to assess dynamic changes in microcirculation in conjunction with StO_2_ monitoring [35]. VOT provides an ischemic challenge to peripheral tissues allowing analysis of tissue response when occlusion is released. The VOT was used in healthy volunteers to examine the relationship between the VOT and StO_2_ measurements [35, 36]. Shapiro and colleagues performed VOT on patients with sepsis syndromes and organ dysfunction in emergency department patients [37]. However, the technique for using VOT has not been standardized [36]. In a sample of healthy volunteers, Suhaimi and coworkers [35] reported rapidly inflating the sphygmomanometer to 40 mmHg above the baseline systolic blood pressure (SBP), with inflation lasting over 10–30 seconds, occlusion for 3 minutes, followed by rapid deflation. Gómez et al. [36] reported that, in healthy volunteers, inflation of a blood pressure cuff to >30 mmHg above the base line SBP was maintained for 3 minutes, or until the StO_2_ decreased to a minimal threshold. The period of inflation was reported to be 3-4 seconds, while deflation time lasted <0.5 seconds. Shapiro and colleagues [37] reported using an automated tourniquet with inflation of the blood pressure cuff to 50 mmHg above the SBP with the inflation lasting 3 minutes, after which time the cuff was rapidly removed. In addition, the arm position of the participants was only mentioned in one article [35], while in the study by Gómez et al. [36] the participants were described as resting in a semirecumbent position in a quiet environment. While Suhaimi et al. [35] reported that 93% of the participants were right hand dominant, no mention was made of the use of the dominant or nondominant hand/arm. Gómez and colleagues [36] included using the dominant hand of 15 volunteers during the study. Arm positioning on a table relative to the axillary region was described in the Gómez study [36], but not by the others [35, 37]. Suhaimi and coworkers [35] required the participants not to move the arm, hand, or fingers after a pulse oximetry probe was attached. Suhaimi et al. [35] allowed the subjects to stabilize movement for at least 5 minutes prior to starting the four serial VOT maneuvers. In the fourth VOT, the subjects squeezed a rubber ball between their index finger and thumb until cuff deflation. Therefore, it would appear that metabolic rate may have varied among participants and, subsequently, influenced StO_2_ measurements.

Lima and colleagues examined whether dynamic changes in StO_2_ values may be confounded by factors other than the desired biomarker of thenar muscle oxygenation [9]. Superficial skin cooling was imposed on 8 healthy volunteers while decreases in core temperature were prevented. The VOT protocol was performed prior to, during, and after cooling. The StO_2_ parameters consisted of the resting StO_2_ value and the desaturation and recovery rates of StO_2_. StO_2_ values and StO_2_ recovery rates significantly decreased in spite of preservation of muscle blood flow. These findings suggested that decreases in StO_2_ may mislead the bedside clinician to assume that tissue hypoxia is present, when this change may merely low blood flow to the skin and subcutaneous layers above the muscle capillary beds.

Gruartmoner and colleagues also investigated dynamic changes in StO_2_ measurements, in this case, during weaning from mechanical ventilation in 37 critically ill patients (Table 2, Study 4) [38]. A Vascular Occlusion Test (VOT) was used to measure sequential changes in tissue oxygen saturation, that is, the slopes of change in the rates of deoxygenation (DeO_2_) during cuff inflation and reoxygenation (ReO_2_) during deflation. A NIRS-derived thenar muscle oxygen consumption (nirVO_2_) was calculated using what the researchers describe as the DeOx slope, tracking StO_2_ changes over time. An unsuccessful weaning process was associated with higher increases in StO_2_ deoxygenation rate (DeO_2_) (*P* = 0.04) and in local skeletal muscle oxygen consumption (nirVO_2_) (*P* = 0.04). The increases in the slope of change in the DeO_2_ were interpreted to indicate adverse acute redistribution of blood flow to the lungs during a weaning trial among patients who were unable to wean. The investigators suggest that if the metabolic demand of transitioning from mechanical ventilation to spontaneous ventilation cannot be met by increasing oxygen delivery, due to either limitations in cardiovascular reserve or increased work breathing, then the cardiovascular system addresses these excessive demands by increasing sympathetic tone to maximize cardiac output, while vasoconstriction redistributes blood flow away from the periphery and splanchnic circulation to the respiratory muscles.

Ives and coworkers [19] performed a retrospective analysis of prospective, within- subjects cohort study (*n* = 59) in order to evaluate whether StO_2_ measurements can better predict postoperative surgical site infections (SSIs) when compared to the National Nosocomial Infection Surveillance (NNIS) risk index, which the investigators report has limited accuracy (Table 2, Study 6). They measured StO_2_ preoperatively over the biceps and at the intended incision site and at 12, 24, and 48 h after surgery. There was a significant difference in StO_2_ values at the surgical site between patients who developed an SSI (43% + 18.1) and those who did not (55% + 22.0) at 12 and 48 hours after operation (*P* = 0.032; *P* = 0.030). While this study was adequately powered* a priori*, well designed, and controlled with baseline measurements and a blinded outcome assessment, the StO_2_ values had large standard deviations with significant overlap.

### 3.7. Studies of Open-Heart Surgical Patients

Putnam and coworkers conducted a prospective, controlled study of sequential changes in multiple variables reflecting oxygen transport parameters, including cardiac output, mean arterial pressure, lactate, base deficit, and StO_2_ (Table 2, Study 12) [39]. The primary finding was that StO_2_ decreases preceded maximal changes in serum lactate by an average of 94 minutes. In a more rigorous study of 74 open-heart surgical patients, with a sample size based on an* a priori* power analysis, Sanders and researchers collected data at the time of induction of anesthesia at a rate of 1/min, during first 20 minutes in ICU and at 2, 6, and 12 hours after surgery until extubation in the ICU or completion of total monitoring time of 24 hours (Table 2, Study 13) [40]. Postoperative morbidity was measured with the Postoperative Morbidity Survey (POMS). The investigators reported that mean StO_2_ measures during the first minutes of anesthesia and at 20 minutes in the ICU were lower in patients with postoperative morbidity scores than in patients without such morbidity on day 3 (72.9% versus 85.5%; *P* = 0.009) and day 15 (81.1% versus 87.6%; *P* = 0.04). While this study was supported by a grant from the manufacturer, the authors do not report whether the manufacturer had access to the data prior to publication.

## 4. Discussion

At the most fundamental level, the adequacy of tissue oxygenation is defined as the balance between the supply of and the demand for oxygen required for cellular function and metabolism at the cellular, organ, and systems levels. When tissue perfusion falls below the critical point at which blood flow to individual organs can no longer be maintained, shock is diagnosed by the progressive onset of hypotension [7, 41]. Bedside clinicians assess end-organ tissue perfusion in critically ill patients by signs of altered mental status, peripheral skin color and warmth, capillary refill, pulse strength, mean arterial pressure, urinary output, and percent oxygen saturation of arterial hemoglobin (SaO_2_) in the form of continuous pulse oximetry (SpO_2_). However, these indicators may remain relatively normal during the early stages of shock, despite the fact that the patient's condition is deteriorating.

Although there is no gold standard for the clinical diagnosis of shock, increased serum lactate and lactate clearance continue to function as indicators of, respectively, the severity of tissue hypoxia and the adequacy of resuscitation from shock [41]. Yet many conditions of elevated lactate levels are not associated with anaerobic metabolism, including epinephrine administration, respiratory and metabolic alkalosis, and ethylene glycol intoxication; in addition, the physiological functions of recycled lactate at the cellular level are beneficial to the viability of red blood cells, cardiac muscle, striated muscle, astrocytes, and neurons [41]. Other conditions, such as diabetic ketoacidosis, impaired hepatic function, and large quantities of exogenous lactate used for resuscitation of traumatic injuries may also lead to elevated lactate levels [42]. In the healthy individual, the heart is a consumer of lactate up to maximal levels of myocardial oxygen consumption [43]. Bakker and colleagues propose that it would be more accurate to use the term* lactate associated metabolic acidosis* to describe the presence of elevated serum lactate levels in the presence of an abnormal arterial pH. Bakker and colleagues point out that although there is only a weak, but statistically significant, relationship between arterial pH and serum lactate levels, the combined presence of metabolic acidosis and elevated serum lactate levels carry a high prognostic indicator of mortality [41].

## 5. Conclusion

The long-studied methodology of near-infrared spectroscopy (NIRS) has recently been applied in a novel technology designed to measure skeletal muscle tissue perfusion in critically ill patients. The basic assumption underlying this clinical application is that when tissue hypoxia occurs, the compensatory vasoconstriction that occurs in peripheral tissues will be signaled by a decrease in the StO_2_. Lima and colleagues emphasize that the peripheral circulation will be the first region to manifest tissue hypoperfusion and the last to show signs of reperfusion following resuscitation [6]. The research presented recommends that a cutoff StO_2_ value of less than 70% to 75% would represent a cause for concern as well as the need for troubleshooting for possible causes of reduced tissue perfusion. However, none of the study designs presented here have utilized randomized sampling, a control group, or sought to establish a cause-and-effect relationship between an intervention aimed at maintaining normal StO_2_ values and specific patient outcomes. Given that investments in new technology in intensive care unit budgets should be evidence based, more research is required using a consistent strategy for interventions in specific patient populations. In addition, we recommend that investigators clarify whether data was shared with the manufacturer of devices prior to publication. As with studies of new technical devices tested in clinical research, the studies under review were characterized by reasonable and expected limitations in design. These initial observational findings show modest promise, providing a necessary step in evaluating the clinical utility of a device designed to monitor a complex phenomenon of tissue oxygenation and its possible use in predicting complications and early identification of patients at risk for complications.

## Figures and Tables

**Table 1:** AACN's new evidence-leveling system.

Level	
A Meta-analysis of multiple controlled studies or metasynthesis of qualitative studies with results that consistently support a specific action, intervention, or treatment.	
B Well-designed controlled studies, both randomized and nonrandomized, with results that consistently support a specific action, intervention, or treatment.	
C Qualitative studies, descriptive or correlational studies, integrative reviews, systematic reviews, or randomized controlled trials with inconsistent results.	
D Peer-reviewed professional organizational standards, with clinical studies to support recommendations.	
E Theory-based evidence from expert opinion or multiple case reports.	
M Manufacturer's recommendations only.	

[10].

**Table 2:** 

References	Type of trial/number of subjects	Patient selection	Purpose/method	Results	Recommendations/comments Level of evidence
[27]	Multicenter, retrospective, observational study at 7 level 1 trauma centers. *n* = 359 trauma patients (356 included in final analysis). This study is a post hoc analysis of a prospective study (number 3 in this table).	Nonprobability sampling. Inclusion criteria: patients who(1) had a SBP <90 mm Hg and BD ≥6 mEq/L within 60 minutes of admission; (2) received red blood cell (RBC) transfusions hours within 6 hours of admission; (3) were inured with at least 1 of the following: fractures (pelvic, long bone, multiple rib), pulmonary contusion, major blunt, or penetrating torso trauma. Exclusion criteria: GCS ≤ 4, cardiac arrest before or upon arrival, advanced directives limiting aggressive care, penetrating or nonsurvivable brain injury, and bilateral upper extremity fractures.	*Determine the relationship between hypothermia and patient outcome.* Hypothermia defined as <35°C within the first 6 hours of admission. Outcome variables at 28 days of hospitalization: (1) multiple organ dysfunction syndrome (MODS); (2) mortality.	Overall incidence of early hypothermia was 43% (155/359). Multivariate logistic regression analysis determined that the minimum StO_2_ (<75%) within the first hour of admission was one of several significant predictors of MODS (*P* = 0.0014) and mortality (*P* = 0.0021) in both normothermic and hypothermic patients. Base deficit was able to predict mortality (*P* = 0.0454), but not MODS, among both hypothermic and normothermic patients.	*Study Strengths*Inclusion and exclusion criteria were clearly specified. Prolonged data collection period. Multicenter sampling increases the external validity of the findings. *Study Limitations * (1) The retrospective nature and post hoc analysis of the study design limit the internal validity of the study. (2) Study funded by the manufacturer. (3) Authors do not disclose whether data was shared with the manufacturer prior to submission of paper. *Level of Evidence*. C
[4]	Prospective observational design. *n* = 24 adults.	Convenience sampling. *Inclusion Criteria*(a) Injury Severity Score (ISS) of at least 25; or (b) ISS = 15, plus need to transfusion of >6 units packed red blood cells (RBCs) within 6 hours of admission. *Exclusion Criteria * Glasgow Coma Scale <8.	*To determine whether there is a relationship between early supply independent mitochondrial dysfunction and multiple organ dysfunction syndrome.* All patients received the same resuscitation protocol with a specific goal for oxygen delivery (DO_2_) of 600 mL/min/m^2^: (1) SaO_2_ > 90%; (2) pulmonary artery wedge pressure (PAWP) 15–18 mm Hg; (3) hematocrit (hct) 35%. Serum lactate sampled at baseline and postresuscitation at 12 hours of admission. Near-infrared spectroscopy (NIRS) was used to measure tissue HbO_2_ and cytochrome a, a3. An additional fiberoptic probe was used to supply infrared light for absorption spectrum of cytochrome a, a3. The definition of mitochondrial oxygen consumption dysfunction, referred to as *decoupling* between tissue HbO_2_ and a, a3, was operationalized as the “absolute rate of change of the cytochrome a, a3 redox state relative to that of HbO_2_ was >0.03 absorbance units per hour” (p. 534).	The primary outcome variable was the incidence of multiple organ failure (MOF). MOF scores obtained within the first 48 hours of admission were not included in the data analysis. At 12 hours within admission, lactate levels were significantly higher in 9 (38%) who developed MOF. Evidence of decoupling was identified by NIRS in 8 (89%) of patients who developed MOF. Supranormal levels of DO_2_ and VO_2_ did not discriminate between patients who did and did not develop MOF, although, in clinical terms, the rates for both were considerably higher in patients who did not develop MOF.	*Study Strengths*Two of the investigators were blinded during the coding of the NIRS data. The interrater agreement was reported to be high (K = 1.0, CI 0.61, 1.00). *Study Limitations* (1) Small sample size. (2) Two authors were employed by the manufacturer. (3) In addition to grants from the National Institutes of Health and a Center of Excellence Award from the Emergency Medicine Foundation, the manufacturer provided a grant for this study. (4) The authors did not report evidence for an objective method for ensuring resuscitation protocols were consistently followed. (5) The authors do not report when MOF scores were obtained. *Level of Evidence*. C
[7]	Multicenter, observational study at 7 level 1 trauma centers. A power analysis based on a 14% incidence of MODS estimated that sample should consist of at least 278 patients in order to achieve a sample size of 35 patients who would likely develop MODS. *n* = 383 patients; 381 included in final analysis.	Nonprobability sampling. Inclusion criteria: patients who (1) had a SBP <90 mm Hg and BD ≥6 mEq/L within 60 minutes of admission; (2) received red blood cell (RBC) transfusions hours within 6 hours of admission; (3) were inured with at least 1 of the following: fractures (pelvic, long bone, multiple rib), pulmonary contusion, major blunt, or penetrating torso trauma. Exclusion criteria: GCS ≤ 4, cardiac arrest before or upon arrival, advanced directives limiting aggressive care, penetrating or nonsurvivable brain injury, and bilateral upper extremity fractures.	(1) *Evaluate predictive capability of StO*_2_ * monitoring for MODS or 28-day mortality in severely injured patients.* (2) *Identify threshold StO*_2_ * value for predicting MODS.* StO_2_ sensor was placed within 30 minutes of ED arrival and remained in place for 24 hours or until death or discharge, if earlier. The minimum StO_2_ value within the first hour of ED arrival was used as the primary variable for data analysis.	MODS: when the minimum cutoff StO_2_ value is set at 75% within the first hour of admission: (a) StO_2_ sensitivity to MODS: 78%. (b) StO_2_ specificity for MODS: 39%. (c) StO_2_ positive predictive value for MODS: 18%. (d) StO_2_ negative predictive value for MODS: 91%. Mortality: when the minimum cutoff StO_2_ value is set at 75% within the first hour of admission: (a) StO_2_ sensitivity to mortality: 91%. (b) StO_2_ specificity for mortality: 37%. (c) StO_2_ positive predictive value for mortality: 20%. (d) StO_2_ negative predictive value for mortality: 96%. StO_2_ predicted which patients will not develop MODS, but poorly in those who do develop it. A minimum StO_2_ of 75% performed significantly better than SBP >90 mm Hg (39% versus 32%) in predicting MODS.	*Study Strengths*There were no adverse events related to the use of the monitor. Investigators were blinded to the StO_2_ data. Power analysis performed *a priori.* *Study Limitations * (1) No standardized resuscitation protocol reported. As a result, physician treatment bias may confound results. (2) The positive predictive values for both MODS and mortality are poor. (3) Authors state that, due to limitations in the availability of monitors and research staff at all hours, the study sample was a “random sample” of consecutive patients. The method of randomization is not stated. (4) StO_2_ collected on the thenar eminence adjacent to the site of radial artery cannulation in 34 patients was removed from the data analysis. (5) Study funded by the manufacturer. (6) Authors do not disclose whether data was shared with the manufacturer prior to submission of paper. *Level of Evidence*. C
[38]	Prospective observational study, *n* = 37.	Adult patients receiving mechanical ventilation for greater than 48 hours and considered ready to wean by physician: (a) partial or complete recovery from the underlying cause of acute renal failure; (b) adequate gas exchange, as indicated by partial pressure of arterial oxygen >60 torr; (c) FiO2 < 40%; (d) PEEP less than 5 cm; core temperature <38°C; Hgb greater than 8 grams/dL; (e) no further need for vasoactive and sedative agents. Exclusion criteria: (a) any injury to extremities that could hinder placement of NIRS sensor probe; (b) altered level of consciousness that could lead to central hypoventilation and/or excessive secretions.	*To determine whether S* *tO*_2_ * associated with an ischemic challenge is related to weaning outcome.* The vascular occlusion test (VOT) was performed at the beginning of the weaning trial and at 30 minutes of weaning, providing paired data for changes in StO_2_.	An unsuccessful weaning process was associated with higher increases in StO_2_ deoxygenation rate (DeO_2_) (*P* = 0.04) and in local skeletal muscle oxygen consumption (nirVO_2_) (*P* = 0.04) after a 30-minute weaning trial. In contrast, successful weaning was not associated with these changes. Both groups of patients showed a significant increase in respiratory rate and heart rate, with no other changes in their hemodynamic, respiratory, and oximetry parameters.	Physician decision-maker for extubation had no access to the StO_2_ data. Standardized weaning trial protocol in place. *Level of Evidence*. C
[28]	Prospective observational pilot study. *n* = 28 trauma patients.	Convenience sampling was used to enroll adult patients who were admitted to the ICU after trauma. Inclusion criteria: Abbreviated Injury Score ≥23. Requiring mechanical ventilation Assessed to be adequately resuscitated defined by protocol:(1) mean arterial pressure [MAP] ≥ 70 mm Hg, (2) heart rate [HR] ≥ 110 bpm, (3) base deficit ≤ 2, (4) partial pressure of arterial oxygen (PaO_2_) = 80-I 50 mm Hg. Average time from hospital admission to placement of devices was 10 hours. Exclusion criteria: patients with head injury who required craniotomy and postoperative vasopressor therapy were excluded; patients with spinal cord injuries.	(1) *To evaluate the relationship between StO*_2_ * and PmO*_2_ * implanted probe with other measures of tissue perfusion*; (2) *to determine values of StO*_2_ * and PmO*_2_ * in resuscitated injured patients*; (3) *to identify the relationship between StO*_2_ * and PmO*_2_ * values with subsequent infection and MODS within the first 24 hours of intensive care unit(ICU) admission. * The Licox monitor, equipped with a polarographic tissue probe, was used to measure the partial tissue pressure of oxygen (*PmO*_2_) in 27 patients. The *InSpectra* monitor was used to noninvasively measure StO_2_ in 25 patients.	The relationship between StO_2_ and PmO_2_ * *values in the same deltoid muscle bed was statistically significant but weak (*r* = 0.05, *P* < 0.0001). The mean PmO_2_ value in fully resuscitated patients was 34 ± 11 mm Hg. The mean *StO*_2_ * *value in fully resuscitated patients was 63 ± 27%. Low PmO_2_ values (≤25 mm Hg) during the first 24 hours of ICU stay were significantly associated with ICU length of stay, MODS, infection, and mortality (*P* < 0.01), while low StO_2_ values (≤35%) were not associated with these outcomes, with one exception: low StO_2_value (≤35%) persisting for longer than 2 hours on the first day of admission was able to predict MODS (odds ratio 12.8 [CI 1.3–131], *P* = 0.03).	*Study Strengths*Clinical criteria of adequate resuscitation were defined *a priori*. *Study Limitations * (1) Small sample size. (2) Power analysis was not performed to determine adequate sample size. (3) The mean PmO_2_ and StO_2_ values reported in this study differ significantly from those published in other studies. *Level of Evidence*. C
[19]	Retrospective analysis of prospective, within-subjects cohort study,*n* = 59.	Samples size estimated by power analysis. Nonprobability sampling. Inclusion criteria: convenience sample of patients undergoing major abdominal or groin bypass surgery.	*Determine whether S* *tO*_2_ * measurements can predict postoperative surgical site infections (SSIs*). Measured StO_2_ preoperatively over the biceps and at the intended incision site and at 12, 24, and 48 h after surgery. Postoperative StO_2_ measurements were taken through transparent dressings at the surgical site. Blinded to StO_2_ readings, an infection control nurse assessed surgical incisions at 7 and 30 days postoperatively. According to the authors, a noninvasive measure of SSI is needed because the National Nosocomial Infection Surveillance (NNIS) risk index has limited accuracy.	17 (29%) subjects developed SSIs. No statistical difference related to age, sex, body mass index, or triceps skinfold thickness was found between groups. All StO_2_ values increased postoperatively, regardless of postoperative SSI outcome. There was a significant difference in StO_2_ at the surgical site between patients who developed an SSI (43% ± 18.1) and those who did not (55% ± 22.0) at 12 and 48 hours after operation (*P* = 0.032; *P* = 0.030). Sensitivity: 70.6%. Specificity: 76.2%. StO_2_ performed significantly better at predicting SSIs than the NNIS risk index.	*Study Strengths*Innovative, well-designed study controlled with baseline measurements. Blinded outcome assessment. Power analysis performed *a priori*. *Study Limitations * Standard deviations for both groups were large with overlap. The mean values are lower than the mean StO_2_ values reported in the literature. The validity of findings is limited by the retrospective design. Postoperative measurements of surgical-site infections (SSIs) were performed at low frequency. Loaned equipment from manufacturer is acknowledged. Authors do not disclose whether data was shared with the manufacturer prior to submission of paper. *Level of Evidence*. C
[31]	Retrospective observational study, *n* = 42.	Nonprobability sampling. Inclusion criteria: convenience sample of consecutively admitted patients diagnosed with septic shock according to the International Sepsis Forum Definition of Infection in the ICU Consensus Conference (2005).	(1) *Determine the relationship between * *StO*_2_ *values and mortality in patients with septic shock after early resuscitation.* Data collection initiated after optimization of macro hemodynamic variables (MAP, urine output, and ScvO_2_) according to the Consensus Conference recommendations.	Sample analyzed by groups: entire sample, survivors on day 3, and survivors on day 28. Nine (64%) patients whose StO_2_ was <78% did not survive to day 28 when compared to 5 patients who did (*P* = 0.002). Overall, StO_2_ values were significantly lower in nonsurvivors than in survivors (73% [68–82%] versus 84% [81–90%], *P* = 0.02). A receiver operating curve analysis confirmed that StO_2_ was associated with mortality with an area under the curve at 71% (52–91%,*P* = 0.03), whereas elevated lactate levels were not significantly related to mortality.	*Study Strengths*Resuscitation treatment protocol clearly stated. *Study Limitations * (1) Endpoint of optimization of hemodynamic variables was determined but may have been modified by the attending physician. (2) Duration of StO_2_ monitoring is unclear. (3) No intervention is based on unblinded StO_2_ values. (4) Power analysis is not performed. (5) Authors do not refer to conflict of interest. *Level of Evidence*. C

[6]	Prospective observational study, *n* = 22.	Convenience sample. Inclusion criteria: elevated lactate levels (>3.0 mmol/L) upon admission to the ICU; absence of history of peripheral vascular disease.	*To test the hypothesis that persistence of low S* *tO*_2_ * values less than 70% is related to organ failure and mortality*. Early goal-directed therapy (EGDT) based on the Surviving Sepsis Guidelines was used to guide resuscitation. StO_2_ measurements were recorded for 8 hours every 2 hours.	Among the 12 patients who at baseline had low *StO*_2_ values, the 10 patients who were unable to normalize low *StO*_2_ values were more likely to develop MODS and have an increased mortality and persistence of elevated serum lactate (*P* < 0.05).	*Study Strengths*(1) StO_2_ monitoring was performed at baseline and every 2 hours during the first 8 hours following resuscitation. (2) Data collector of NIRS data blinded to treatment and clinical information. *Study Limitations * (1) Small sample size. (2) Publication of this information in a journal supplement was supported by the manufacturer. *Level of Evidence*. C
[32]	Prospective, observational design, *n* = 15.	Inclusion criteria: early stage of septic shock (authors state “less than 24 hours” but do not specify when measurements began); decision to insert PAC catheter; stabilized MAP at least 65 mm Hg for 30-minute period.	(1) *Test the significance of the relationship between * *StO*_2_ * and global DO*_2_.	The correlation between StO_2_ and global DO_2_ was 0.78 (*P* = 0.001). There were also strong statistical correlations with SvO_2_ (0.77, *P* = 0.001), CvO_2_ (0.8, *P* = 0.001), and O_2_ER (−0.76, *P* = 0.002). A cutoff StO_2_ value of 75% predicted an extremely low DO_2_ (<450 mL/min/m^2^) with a sensitivity and specificity of 90%. Serum lactate values among patients with high DO_2_ (4.1 ± 2.5) versus low (5.2 ± 2.9) were clinically but not statistically different. Mean StO_2_ value among patients with high DO_2_ was 84% ± 8. The mean StO_2_ value among patients with a low DO_2_ was 68% ± 5 (*P* = 0.0002). StO_2_ monitoring did not correlate with moderately low DO_2_ (450–600 mL/min/m^2^). There was no relationship between DO_2_ level and mortality. The authors do not report whether there was a significant relationship between StO_2_ levels and mortality.	*Study Strengths*These findings suggest that, in the absence of a PAC catheter, StO_2_ and serum lactate might accurately substitute for DO_2_ measurements. *Study Limitations * (1) Hemodynamic management protocol was not described. (2) Patient recruitment occurred at different points during resuscitation. (3) The authors point out that StO_2_ values did not correlate with moderately low DO_2_, thereby suggesting that StO_2_ has greater sensitivity to only extremely low DO_2_. (4) Small sample size. *Level of Evidence*. C
[33]	Case controls matched to age and sex: 9 healthy volunteers and 10 patients.	Nonprobability sampling. Inclusion criteria: convenience sample of patients admitted to ICU with severe sepsis and one episode of hypotension (SBP < 90 mm Hg) within 24 hours of study entry as well as criteria based on the SCCM/ACCP Consensus Conference (1995): at least one organ dysfunction, positive culture results, and 2 of the following 4: WBC >12,000 or <4000/mm^3^, fever >38 C or <36 C, tachycardia >90/min, and tachypnea >20 breaths/min. Exclusion criteria: patients in whom a PA catheter was contraindicated.	*Determine the magnitude of the relationship between S* *tO*_2_ * measurements and severity of sepsis and invasive hemodynamic measurements. *	Volunteers had higher MAP, lower serum lactate concentration, and higher StO_2_ than patients with sepsis (*P* = 0.031). Near-infrared spectroscopy-derived mixed venous oxygen saturation (NIRSS_v_O_2_) and StO_2_ correlated with SvO_2_ measured by blood drawn from the PA catheter (*r* = 0.267, *P* < 0.05).	*Study Strengths*The correlations between NIRS-related data and SvO_2_ were found to be statistically significant, but weak (*P* = 0.267). Caregivers were blinded to StO_2_ data. *Study Limitations * (1) Small sample size. (2) Relationship to patient outcome was not analyzed. (3) One author was employed by the manufacturer. (4) No statement regarding whether data was shared with manufacturer prior to submission for publication. *Level of Evidence*. C

[34]	Descriptive correlational study, *n* = 8	Nonprobability sampling. Inclusion criteria: septic shock and MODS.	*Determine whether degree of edema confounds accurate measurement of S* *tO*_2_. *InSpectra* probes were placed at multiple muscle sites: bilateral deltoid muscles, brachial and thenar eminence, and bilateral vastus and gastrocnemius muscles. Tissue thickness and edema were measured by echography.	The lowest variance (within-subject variability) of StO_2_ was identified in the thenar eminence (22%). The degrees of edema and skin thickness were lowest in the thenar eminence. StO_2_ values significantly correlated with degree of edema (*r* ^2^ = −0.44, *P* < 0.0001) and with total tissue thickness from skin to muscle *r* ^2^ = −0.64, *P* = 0.0001).	*Study Strengths*Pilot study examining methodological issues of accuracy and precision of StO_2_ measurement. *Study Limitations * (1) Accuracy and precision of echocardiography not reported. (2) Small sample size. *Level of Evidence*. C
[39]	Prospective observational study, *n* = 40	Nonprobability sampling. Inclusion criteria: convenience sample of all adult patients who underwent cardiopulmonary bypass (CPB) procedures.	(1)* To determine the ability of* *StO*_2_ *to detect subtle changes in tissue oxygenation in a controlled model of altered perfusion.* (2)* Compare * *StO*_2_ * with standard hemodynamic variable (MAP and DO*_2_) (3)* Examine the temporal relationship between * *StO*_2_ * and standard biochemical variables in real-time CPB. * Measured vital signs and central pressures at stages of CPB: preinduction, postinduction, sternotomy, initiation of CPB, after 30 minutes of CPB at start of rewarming, when CPB was terminated, and at sternal closure. Data analysis focused on StO_2_, MAP, CO, DO_2_, lactate, BD, and temperature.	StO_2_ changes: initiation of CPB: decreased by 12.9% (*P*, 0.0001); this decrease preceded maximum lactate level by on average 94 minutes; this decrease also correlated with a decrease in DO_2_. StO_2_ increased from the rewarming period to termination of CPB (*P* = 0.0001). A threshold value of 79% was identified as an indicator of hypoperfusion.	*Study Strengths*Perioperative caregivers were blinded to the StO_2_ data. *Study Limitations * (1) A power analysis was not performed to optimal sample size. (2) A table presenting the variables of interest at each stage of surgery would have been helpful in the interpretation of real-time changes in key variables. *Level of Evidence*. C

[40]	Prospective observational study. *n* = 74 adult patients undergoing cardiac surgery.	Convenience sampling. Inclusion criteria: first-time CABG or cardiac valvular surgery requiring cardiopulmonary bypass. Exclusion criteria: patients were excluded for the following circumstances: emergent surgery; congenital heart condition; current participation in a clinical intervention trial; or having dermatological contraindications for use of the *InSpectra *probe.	*To determine the degree of association of changes in tissue oxygen saturation with postoperative outcome in cardiac surgery patients.* *The primary outcome was postoperative morbidity.* Data was collected at the induction of anesthesia at a rate of 1/min during first 20 minutes in ICU and at 2, 6, and 12 hours after surgery until extubation in the ICU or completion of total monitoring time of 24 hours.	Mean StO_2_ measures during the first minutes of anesthesia and at 20 minutes in the ICU were lower in patients with postoperative morbidity scores than in patients without such morbidity on day 3 (72.9% versus 85.5%; *P* = 0.009) and day 15 (81.1% versus 87.6%; *P* = 0.04).	*Study Strengths A priori *power analysis was performed to determine sample size. *Study Limitations * (1) This study was supported by a grant from the manufacturer. (2) The authors do not report whether the manufacturer had access to the data prior to publication. *Level of Evidence*. C
[29]	Prospective, observational study. *n* = 26 trauma patients	Convenience sampling. Inclusion criteria: patients estimated to be at risk for hemorrhagic shock, including any of the following: SBP ≤ 90 mm Hg; injury to neck, chest, abdomen, flank, buttock, or proximal extremities; uncontrollable hemorrhage; or suspected ongoing internal hemorrhage. Exclusion criteria: any of the following: isolated head injury; prehospital traumatic cardiopulmonary arrest; transfers from other hospitals; hand injury preventing placement of StO_2_ sensor.	(1) *Determine the threshold * *StO*_2_ * value predictive of need for blood transfusion.* (2) *Evaluate the potential relationship between * *StO*_2_ * values and hospital length of stay and mortality.* StO_2_ measured for 1 hour after arrival to trauma bay. Independent variable: minimum StO_2_ used as marker for trigger to blood transfusion within first hour of admission (dependent outcome variable).	Sample categorized as: HI-StO_2_ ≥ 70% or LO-StO_2_ < 70%. No statistical differences between 2 groups based on gender, age, ISS, RTS, SBP, HR, serum lactate, BD, hgb, or 30-day mortality were found. LO-StO_2_ patients 9 times more likely to receive transfusion (64%) than HI-StO_2_ group (7%) (*P* = 0.018). Survivor length of hospital stay longer in LO-StO_2_ patients than in HI-StO_2_ patients (*P* = 0.0001).	*Study Strengths*(1) Caregivers blinded to the StO_2_ data. (2) Inclusion/exclusion criteria clearly stated. *Study Limitations * (1) Authors acknowledge that selection bias may have occurred, as decision to treat with transfusion made by attending trauma surgeon or chief surgical resident. (2) Small sample size. (3) Authors do not refer to conflict of interests. *Level of Evidence*. C

StO_2_: tissue oxygen saturation; DO_2_I: oxygen delivery indexed to body surface area; VO_2_I: oxygen consumption determined by metabolic cart, indexed to BSA: body surface area; SvO_2_: mixed venous oxygen saturation; hgb: hemoglobin; hct: hematocrit; ABG: arterial blood gas; NIRS: near-infrared spectroscopy; PA: pulmonary artery; Sensitivity: proportion of actual positives which are correctly identified; Specificity: proportion of negatives which are correctly identified; SSI: surgical-site infection; PAD-IC: peripheral arterial disease with intermittent claudication; ABI: ankle brachial index; BMI: body mass index; MODS: multiple organ dysfunction syndrome; SBP: systolic blood pressure; ED: emergency department; CPB: cardiopulmonary bypass; CO: cardiac output; DO_2_ mL/minute: oxygen delivery measured in milliliters per minute; ISS: Injury Severity Score; RTS: Revised Trauma Score; BD: base deficit; ScvO_2_: central venous oxygen saturation of oxygen; ROC: receiver operating characteristic curves; *T*_50_: time in seconds from peak exercise to 50% StO_2_ recovery; *T*_100_: time elapsed from peak exercise to 100% StO_2_ recovery time.

*Definition of terms is as follows.

Sensitivity: percentage of subjects who have both the condition (MODS) and a positive test.

Specificity: percentage of patients who did not have the condition (MODS) and a negative test.

Positive predictive value: percentage correctly diagnosed with a positive test.

Negative predictive value: percentage correctly diagnosed with a negative test.
